# Influence of Desulfurization with Fe_2_O_3_ on the Reduction of Nickel Converter Slag

**DOI:** 10.3390/ma13102423

**Published:** 2020-05-25

**Authors:** Guangsheng Wei, Yun Wang, Rong Zhu, Lingzhi Yang

**Affiliations:** 1 School of Metallurgical and Ecological Engineering, University of Science and Technology Beijing, Beijing 100083, China; wgshsteel@126.com (G.W.); zhurong12001@126.com (R.Z.); 2 Beijing Key Laboratory of Research Center of Special Melting and Preparation of High-End Metal Materials, University of Science and Technology Beijing, Beijing 100083, China; 3 The China ENFI Engineering Co., Ltd., Beijing 100038, China; 4 School of Minerals Processing and Bioengineering, Central South University, Changsha 410083, China

**Keywords:** nickel converter slag, desulfurization, reduction, Fe_2_O_3_, metal recovery

## Abstract

Generally in the nickel converter slag, metals are mainly in the form of sulfides, which are difficult to separate from slag. Although metal oxides in the slag, such as NiO, CoO, and Cu_2_O, are easily reduced into metal using carbon, the presence of sulfur inhibits the reduction reaction. In this study, the addition of Fe_2_O_3_ to nickel converter slag produced desulfurized slag, which enhanced the carbothermal reduction process. Increasing the desulfurization rate promoted the conversion of sulfides into oxides in slag, which significantly increased the activity of NiO, Cu_2_O, and Fe_2_O_3_. However, the residual sulfur content had no significant effect on the activity of FeO and CoO, due to the high initial FeO content and cobalt existing mainly in the form of oxides. The optimum addition of Fe_2_O_3_ was 15.0 g per 100 g nickel slag, while the desulfurization ratio was 36.84% and the rates of nikel, cobalt and copper recovery were 95.33%, 77.73%, and 73.83%, respectively.

## 1. Introduction

The process of oxidizing low-nickel matte using flowing air in a horizontal converter results in a large amount of slag containing nickel, cobalt, copper, and other metals. A subsequent process is used to clean the nickel slag in order to extract some of the nickel, cobalt, and copper [1,2,3]. The molten slag is poured into the melting pool of an electric furnace; then, a matte with high sulfur content is produced by adding coal and pyrite [4,5]. However, the treatment cycle is long and gives poor dilution results of residue copper up to 0.5–1.0% [6,7]. According to previous studies [8], the main components of nickel converter slag is FeO and SiO_2_, where Ni mainly exists as sulfides and some distributes in fayalite or composite oxides. Cobalt is mainly dispersed in fayalite and most of the copper exists as sulfides [9,10]. Therefore, in the nickel converter slag, the sulfides of Ni and copper result in the loss of Ni and Cu [11,12].

It is of great importance to separate the matte from the slag for the recovery of valuable metals and some key performance parameters determines the separation of matte and slag, including density, viscosity and interfacial tension. Various studies have been carried out to investigate the matte and slag’s interfacial tension and density in alloy–slag system [13,14,15,16,17,18,19,20]. For molten slag systems, the density values of FeO_x_-SiO_2_-Al_2_O_3_ [13], FeO_x_-SiO_2_-MgO [13] and FeO_x_-SiO_2_-CaO-Al_2_O_3_ [14] are in the range of 3.68~3.69 g/cm^3^, 3.61~3.69 g/cm^3^ and 2.794~2.836 g/cm^3^ respectively. For molten matte systems, the density values of Fe-Ni-S [13], Ni-Cu-S [13] and Fe-Cu-Ni-S [15] are in the range of 3.82~5.18 g/cm^3^, 5.18~5.25 g/cm^3^ and 3.92~5.59 g/cm^3^ respectively. For molten metal systems, the density values of Ni-Cu-Fe [16], Fe [17], Ni [17] and Co-Cu-Fe [18] are in the range of 7.1~8.0 g/cm^3^, 6.905~7.25 g/cm^3^, 7.60~7.73 g/cm^3^ and 7.22~7.62 g/cm^3^ respectively. As for the interfacial tension between molten slag and matte, the values of Fe-Ni-S & FeO_x_-SiO_2_-Al_2_O_3_, Fe-Ni-S and FeO_x_-SiO_2_-MgO are in the range of 0.005~0.180 N/m, 0.026~0.192 N/m [13]. And for that between molten slag and metal, the values of Fe-Ni and CaO-SiO_2_-Al_2_O_3_ is in the range of 0.960~1.670 N/m [19,20]. It can be found that the distribution range of the density of slag is comparatively close to that of the matte while the interfacial tension between matte and slag is particularly small. Hence, the matte can be easily dispersed in the molten slag and it is difficult to separate the matte from the slag. Conversely, it is obvious that the density of the metal with lower sulfur is much larger than that of slag and the value of the interfacial tension between metal and slag is much higher, which indicates that metals are easy to separate from slag using density separation methods. For the nickel converter slag, if the reduction product after desulfurization in the electric furnace is metal rather than matte, a metal containing large amounts of nickel, cobalt, and copper can more easily be separated from the slag. The resulting metal has lower sulfur content than the matte, which is achieved by first removing sulfur and then reducing the metal oxides in the slag.

CaO was used to remove the sulfur for molten metal by forming CaS in the molten metal with low sulfur content [21,22,23]. And with high sulfur content, the sulfur was removed as SO_2_ with the method of gasification desulfurization [24,25,26]. Li et al. [27] found that high sulfur content of molten iron would cause the generated iron worthless when using the direct smelting reduction method. Huang et al. [28] and Hu et al. [29] studied the effect of the secondary refining on the removal of sulfur from the slag. Li et al. [30] studied the process the smelting oxidation-reduction process of copper slags with air blowing. And results show that the sulfur content can be reduced from 0.52 wt.% to less than 0.01 wt.% with this method. However, it should be noted that blowing air would lead to the peroxidation of the molten slag and as a result, a large amount of reductant would be consumed and the smelting time is increased. Therefore, it is necessary to utilize weak oxidant to remove sulfur firstly in the recovery of valuable metals from the nickel converter slag through oxidation and reduction. The method of Fe_2_O_3_ injection was proposed for desulfurization before the reduction of nickel converter slag because Fe_2_O_3_ is a weak oxidant and it has an oxidizing effect on slag theoretically. However, few theoretical and experimental studies of the effect of Fe_2_O_3_ on the desulfurization of nickel converter slag. What’s more, metal oxides such as NiO, CoO, and Cu_2_O are easily reduced into metals, while sulfur in the slag cannot participate in the reduction reaction [31,32]. For example, NiO is extremely prone to reduction by CO, while Ni_3_S_2_ is of great difficulty to reduce, similar to the behavior of Cu_2_O and Cu_2_S [33,34,35,36]. The removal of sulfur decreases the ratio of sulfides to oxides, which increases the activity of oxides and promotes the reduction reaction and formation of metals.

In this study, theoretical analysis and tubular furnace experiments were carried out to investigate the effect of desulfurization with Fe_2_O_3_ on the smelting oxidation-reduction process of nickel converter slag. The Fe_2_O_3_ was used to remove the sulfur from the molten slag with sulfur content being 1.53 wt.%. And then carbon was applied to deal with the desulfurized slags with different sulfur contents. The thermodynamic software program FactSage 7.0 was used to simulate the process of slag oxidation, and the simulated data were compared with experimental results. In addition, the desulfurization rate and recovery of valuable metal was studied.

## 2. Thermal State Experiment

### 2.1. Materials

In this study, the nickel converter slag from Jilin Jien Nickel Industry smelter factory (Jilin, China) was adopted. And the method of chemical analysis was applied to test the raw materials and slag and metals from the experiment. As reported by previous studies [8,9,10,11,37], nickel mainly exists as sulfides and some distributes in fayalite or composite oxides, cobalt is mainly dispersed in fayalite and copper mainly exists as sulfides. The elemental distribution of nickel, cobalt, and iron in nickel converter slag is dispersive and uniform. In this study, the remaining moisture was removed by drying the sample slag for 6 h at 110 °C. The slag was processed into powder and the ratio of powder that being less than 100 mesh is larger than 95%. Table 1 lists the chemical composition of nickel converter slag. The chemical compositions of the nickel converter slag are 3.51 wt.% Ni, 0.60 wt.% Co, 0.92 wt.% Cu, 1.53 wt.% S, 43.2 wt.% Total Fe, 52.8 wt.% FeO, 29.2 wt.% SiO_2_, 0.11 wt.% CaO, 0.83 wt.% MgO, and 0.48 wt.% Al_2_O_3_; the ferric oxide (Fe_2_O_3_ > 99.0 wt.%) was applied as the oxidizer; and carbon powder (C > 99.85 wt.%) was applied as the reducing agent.

### 2.2. Experiments

**Figure 1:**
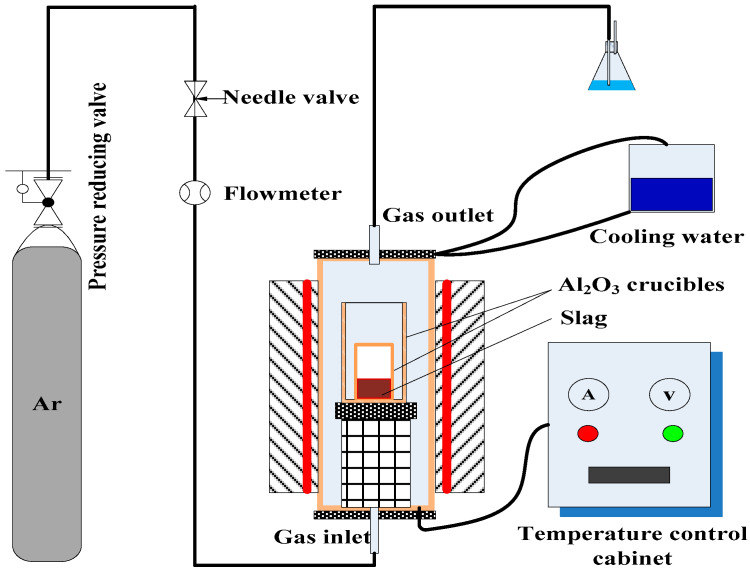
Schematic of the experimental equipment.

Figure 1 presents the thermal experiment flat platform used in this study, including a tubular furnace (Zhongkebeiyi, Beijing, China) with Ar protective gas. The sample was placed in an alumina crucible (φ40 mm × 90 mm), and held in an alumina safety crucible (φ60 mm × 150 mm) to protect the furnace tube from inner crucible failure. The furnace temperature was increased to 1000 °C at a rate of 10 °C/min. Then, the crucible containing the sample was placed in the tubular furnace at 1000 °C. The furnace was resealed and the furnace temperature was further increased from 1000 to 1300 °C at a rate of 5 °C/min; then the temperature was maintained at 1300 °C for desulfurization or reduction. The slag and metal obtained from the experiments were analyzed in Testing Center of University of Science and Technology Beijing (USTB).

Table 1 shows the experimental schemes for the desulphurization of initial nickel converter slag with Fe_2_O_3_. The Fe_2_O_3_ (0–40.0 g) and the nickel converter slag (100.0 g) were mixed in the desired ratios and these mixtures were held at 1300 °C for 30 min. Then, the molten slag was cooled in flowing Ar. Half of the cooled desulfurized slag was applied in the subsequent reduction experiments, while the remainder was used for analysis by a SPECTRO X-ray Fluorescence Spectrometer, SPECTRO Analytical Instruments, Kleve, Germany.

Table 2 shows the experimental schemes for the reduction of the desulfurized slag with carbon powder. In this study, the carbon powder was added with the C/O ratio being at 0.2. The desulfurized slag (~50–70 g) and the carbon powder were mixed, and the mixture was heat treated at 1300 °C for 60 min. After reduction and separation, the sample was cooled in flowing argon. The metal formed in the reduction experiments were sent for analysis.

The FactSage 7.0 thermodynamics software (7.0, FactSage, GTT-Technologies, Herzogenrath, Germany) was used to characterize the chemical equilibrium of the reactions during the desulfurization process, and the activities of FeO, Fe_2_O_3_, NiO, CoO, and Cu_2_O during this process were calculated. According to previous research [37], the ratios of Ni, Co, and Cu existing as sulfide were 51.64%, 15.33%, and 90.08%, respectively. It was assumed that Equation (1) the ratios of Ni, Co, and Cu existing as sulfide were, respectively, 50%, 15%, and 90% and Equation (2) all other sulfur in the slag existed as FeS. The input data used for simulations with the Equilib module of FactSage were 2.23 wt.% NiO, 2.39 wt.% Ni_3_S_2_, 0.65 wt.% CoO, 0.14 wt.% CoS, 0.10 wt.% Cu_2_O, 1.04 wt.% Cu_2_S, 3.00 wt.% Fe_2_O_3_, 51.40 wt.% FeO, 1.76 wt.% FeS, 29.20 wt.% SiO_2_, and 8.09 wt.% Al_2_O_3_ (The total slag weight was 100 g, and other substances in the slag were supplemented using Al_2_O_3_).

## 3. Results and Discussions

### 3.1. Liquidus Temperature of Desulfurized Slag

**Figure 2:**
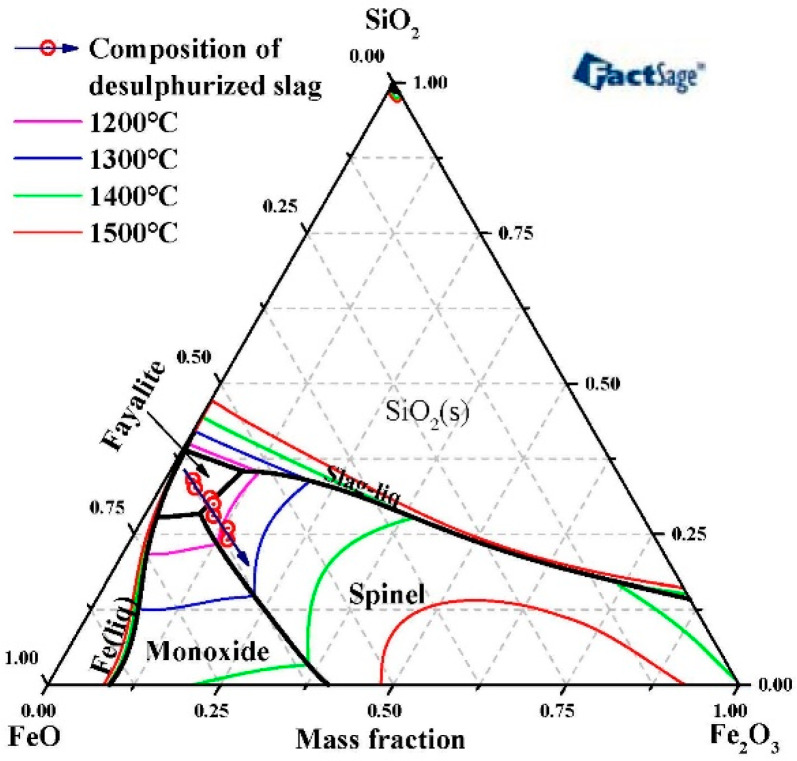
Phase diagram of slag: desulphurization slag composition in the ternary phase diagram of FeO-Fe_2_O_3_-SiO_2_ (obtained from FactSage).

In the nickel converter slag, the total content of FeO_x_ and SiO_2_ was larger than 80% and the liquidus temperature of slag was determined using FeO-Fe_2_O_3_-SiO_2_ phase diagram [38,39,40,41]. Although the increase in the slag liquidus temperature had almost no influence on the desulfurization and reduction progress, it is helpful to separate the metal from the slag with the slag with a lower liquidus temperature [42]. According to the previous study [37], it can be known that the addition of Fe_2_O_3_ influences the liquidus temperature of the slag significantly. The liquidus temperature of slag can be reduced when the Fe_2_O_3_ addition being less than 6 wt.% and that would be increased when the Fe_2_O_3_ addition in the range of 6–40 wt.%. According to the composition of total Fe and FeO in desulfurized slag in Table 2, the content of FeO, Fe_2_O_3_ and SiO_2_ can be calculated. The composition of the desulfurized slag in the ternary phase diagram of FeO-Fe_2_O_3_-SiO_2_ is shown in Figure 2. With the addition of Fe_2_O_3_, the composition of slag moves towards the region with high liquidus temperature.

### 3.2. Equilibrium of Sulfide and Oxide in Slag

Although Fe_2_O_3_ has weaker oxidizing performance than O_2_, the added Fe_2_O_3_ can react with the sulfides (Ni_3_S_2_, CoS, Cu_2_S, and FeS) in the slag. The reactions are as follows:

7Fe_2_O_3_ + Ni_3_S_2_ = 14FeO + 3NiO + 2SO_2_ (g) (1)

(2) $$\Delta G_{Ni}=\Delta G_{Ni}^{\theta }+RTln\frac{a_{FeO}^{14}\cdot a_{NiO}^{3}\cdot {\left(p_{SO_{2}}/p^{\theta }\right)}^{2}}{a_{Fe_{2}O_{3}}^{7}\cdot a_{Ni_{3}S_{2}}}$$

3Fe_2_O_3_ + CoS = 6FeO + CoO + SO_2_ (g) (3)

(4) $$\Delta G_{Co}=\Delta G_{Co}^{\theta }+RTln\frac{a_{FeO}^{6}\cdot a_{CoO}\cdot {(p}_{SO_{2}}{/p}^{\theta })}{a_{Fe_{2}O_{3}}^{3}\cdot a_{CoS}}$$

3Fe_2_O_3_ + Cu_2_S = 6FeO + Cu_2_O + SO_2_ (g) (5)

(6) $$\Delta G_{Cu}=\Delta G_{Cu}^{\theta }+RTln\frac{a_{FeO}^{6}\cdot a_{Cu_{2}O}\cdot {(p}_{SO_{2}}{/p}^{\theta })}{a_{Fe_{2}O_{3}}^{3}\cdot a_{Cu_{2}S}}$$

**Figure 3:**
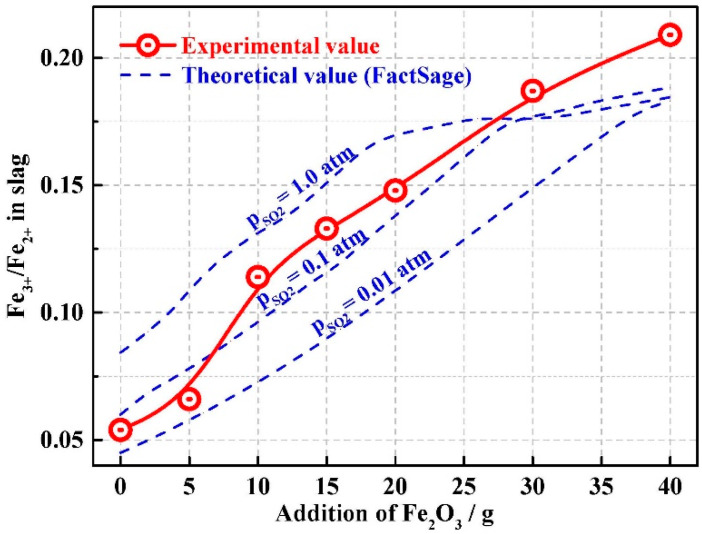
Variation of slag oxidation (Fe^3+^/Fe^2+^) with Fe_2_O_3_ addition.

3Fe_2_O_3_ + FeS = 7FeO + SO_2_ (g) (7)

(8) $$\Delta G_{Fe}=\Delta G_{Fe}^{\theta }+RTln\frac{a_{FeO}^{7}\cdot {(p}_{SO_{2}}{/p}^{\theta })}{a_{Fe_{2}O_{3}}^{3}\cdot a_{FeS}}$$

where $\Delta G_{M}$ (M = Ni, Co, Cu, and Fe) is the Gibbs free energy (J/mol) of the chemical reactions, $\Delta G_{M}^{\theta }$ is the Gibbs free energy in the standard state, R = 8.314 J/(mol·K) is the universal gas constant, T is temperature (K), *a_N_* is the activity of N (N = Fe_2_O_3_, FeO, NiO, CoO, Cu_2_O, FeS, Ni_3_S_2_, CoS, and Cu_2_S) in the slag, $p_{SO_{2}}$ is the partial pressure of gaseous SO_2_ (Pa), and *p^θ^* is the standard atmospheric pressure, 101325 Pa. When the desulfurization reaction reaches equilibrium, Δ*G* = 0. The ratio of the sulfides to oxides in the slag changed gradually during oxidation. At constant temperature conditions (1300 °C), the distribution ratio depends on the composition of the melting slag (NiO, FeO, and Fe_2_O_3_ contents) and the SO_2_ partial pressure ($p_{SO_{2}}$). Based on the above reactions, it can be found that a high Fe^3+^/Fe^2+^ ratio, resulting in a low $\frac{a_{FeO}{}^{2}}{a_{Fe_{2}O_{3}}}$ ratio and low p_SO_2__, are beneficial for converting sulfides to oxides. This agreed with previous reports [11,15]. The ratio of Fe^3+^/Fe^2+^ is mainly influenced by the amount of added Fe_2_O_3_, while $p_{SO_{2}}$ is mainly influenced by the rate of SO_2_ release during the reaction. Figure 3 shows that the ratio of Fe^3+^/Fe^2+^ in the slag measured experimentally increased with increasing Fe_2_O_3_ content, consistent with the theoretical calculations. In this paper, the equilibrium value of sulfur in slag has a balance relationship with SO_2_ concentration in the gas phase. When Fe_2_O_3_ was used for desulfurization reaction in the tubular furnace, the release rate of SO_2_ is different under different conditions, but the flow rate of argon is not constant all the time, which will cause a great difference in SO_2_ concentration in the gas phase. Therefore, results with different SO_2_ partial pressure were calculated by Factsage calculation as shown in Figure 3. What’s more, When more Fe_2_O_3_ was added (30 g, 40 g), more Fe_2_O_3_ will not participate in the reaction of sulfide oxidation, and the utilization rate of oxidant is low, so that the Fe^3+^ remaining in slag is higher, causing the curve to deviate from the calculated value of Factsage calculation.

### 3.3. Desulphurization Rate

In order to compare the influence of Fe_2_O_3_ addition on sulfur removal, the desulfurization rate was defined as:

(9) $$\varphi =1-\frac{m\cdot w\left(S\right)}{m_{0}\cdot w_{0}\left(S\right)}\times 100\%$$

where *φ* is the desulfurization rate (%), *m* is the mass of the slag after the desulfurization (g), *m*_0_ is the mass of initial nickel converter slag (g), *w*(*S*) is the mass fraction of sulfur in the desulfurized slag (%), and *w*_0_(*S*) is the mass fraction of sulfur in the initial nickel converter slag (%).

**Figure 4:**
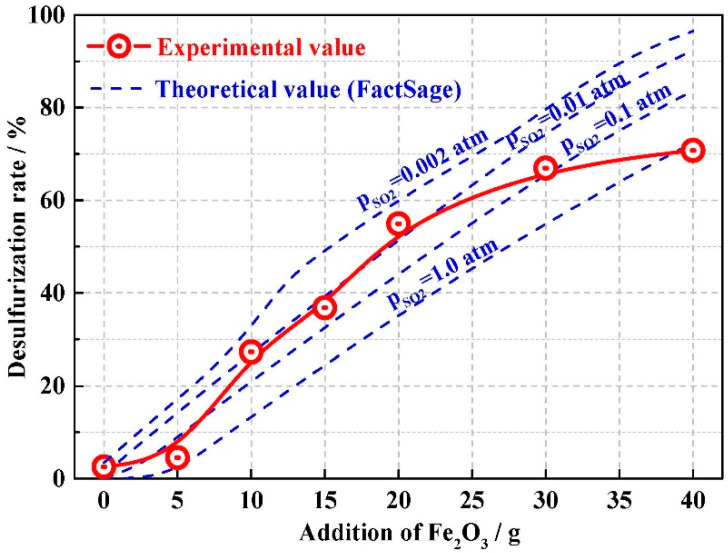
Effect of Fe_2_O_3_ addition on desulfurization rate.

Figure 4 shows the relationship between the added Fe_2_O_3_ and the desulfurization rate for both experimental and theoretical results. When a small amount of Fe_2_O_3_ was added (0–5 wt.%), the desulfurization rate was relatively low after reaction. In this case, the initial Fe_2_O_3_ content in the slag was low, and most of added Fe_2_O_3_ was dissolved into the slag instead of participating in the desulfurization reaction. When 10–20 wt.% Fe_2_O_3_ was added, the slag was strongly oxidized, where the sulfur in the slag was oxidized to SO_2_. Hence, the desulfurization rate increased significantly with the addition of Fe_2_O_3_, as shown in Figure 4. With further addition of Fe_2_O_3_ (20–40 wt.%), the rate of increase of the desulfurization rate decreased. Under these conditions, the sample could not reach equilibrium within the limited time of the experiment due to the high viscosity of the slag. Therefore, the desulfurization rate increased slowly and inadequate contact between S^2−^ and Fe_2_O_3_ in the slag was the main limiting factor for the desulfurization reaction [43]. In addition, the SO_2_ concentration of the product in the reaction process will affect the sulfur content of the equilibrium slag, while the SO_2_ concentration in the gas phase will be different due to the different SO_2_ release rate in the oxidation desulfurization reaction during the experiment process, which may affect the different desulfurization process. The Factsage calculation can quantify and compare the equilibrium value under different SO_2_ partial pressure with the test results.

### 3.4. Activity of Metal Oxide in Slag

Ni, Co, and Cu mainly existed as NiO, CoO, Cu_2_O, Ni_3_S_2_, CoS, and Cu_2_S in the slag. The gaseous products of oxides reduced by carbon are mainly CO, while the gaseous products of sulfide reduction are mainly CS_2_ [38]. According to our previous study [37], the Standard Gibbs free energy of relative reactions have been obtianed from thermodynamic modeling using FactSage. Considering the change in the Gibbs free energy, carbon reacts most readily, first with metal oxides and then with sulfides, in the order of Cu_2_O, NiO, CoO, FeO, Fe_2_SiO_4_, CoS, Ni_3_S_2_, FeS, and Cu_2_S. The ability to oxidize the slag has an equilibrium relationship with the sulfur content in the desulfurized slag, which further influences the ratio of metal sulfides to oxides. According to our previous study [37], the predominant reactions were the reduction of metal oxides by carbon and the increases in the activities of FeO, Fe_2_O_3_, NiO, CoO, and Cu_2_O in the slag could promote the reduction reaction.

**Figure 5:**
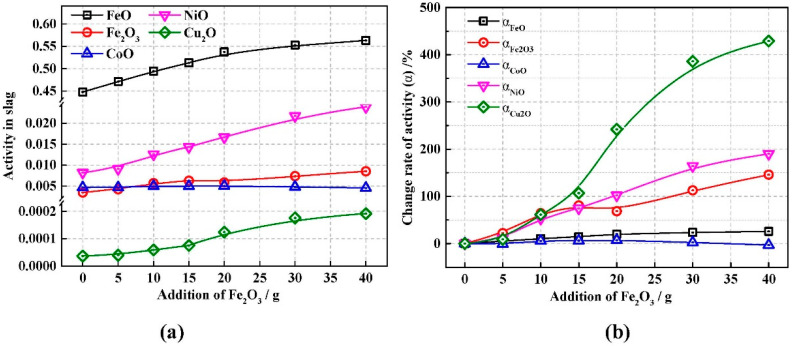
Addition of Fe_2_O_3_ versus (**a**) activity of oxides, and (**b**) activity change rate of oxides.

As shown in Figure 5a, the activities of FeO, Fe_2_O_3_, NiO, CoO, and Cu_2_O in desulfurized slag were exported from FactSage to analyze the influence of desulfurization rate on the activity of metal oxide in the desulfurized slag. With the addition of Fe_2_O_3_, the sulfur content decreased and the activities of FeO, Fe_2_O_3_, NiO, and Cu_2_O increased, while the activity of CoO was almost not affected by the increase of Fe_2_O_3_ addition.

In order to compare the relative changes in activity, the activity of the oxides changes with the amount of Fe_2_O_3_ addition was defined as:

*α* = (*a* − *a*_1_)/*a*_1_ × 100% (10)

where *a* is the activity of oxides in Experiment No. 1–7; *a*_1_ is the activity of the oxides after desulfurization in Experiment No. 1.

With the addition of Fe_2_O_3_, the increase of activity in Cu_2_O, NiO, and Fe_2_O_3_ were significantly, while the increase of activity in FeO and CoO were not. Although Fe_2_O_3_ was added, the most notable increase in activity was for Cu_2_O, rather than Fe_2_O_3_ or FeO, according to Figure 5b. The added Fe_2_O_3_ reacted with sulfides, such as Ni_3_S_2_, CoS, and Cu_2_S, where the sulfur was removed in the form of SO_2_. After sulfur removal from the slag, sulfides in the slag were converted into oxides, increasing the amount (and activity) of metal oxides in the slag. According to the analysis of the existing forms of nickel, cobalt, and copper in nickel slag [44], copper exists mainly in the form of sulfide, which makes sulfur removal have the most significant effect on copper oxide and sulfide in the slag. However, cobalt mainly exists in the form of oxide, and its activity was barely affected by sulfur in the slag. Although most of the added Fe_2_O_3_ was oxidized to FeO, the Fe^2+^ in the slag increased, while Fe^3+^/Fe^2+^ increased simultaneously. Because of the high initial FeO content, the increase in Fe^3+^ fraction was larger, i.e., ΔFe^3+^/Fe^3+^ was greater than ΔFe^2+^/Fe^2+^ (ΔFe^3+^ and ΔFe^2+^ are the increase in the Fe^3+^ and Fe^2+^ ion concentrations in the slag, respectively). This resulted in a larger rate of change in activity for Fe_2_O_3_ than FeO, as shown in Figure 5b.

### 3.5. Recovery of Nickel, Cobalt, and Copper

**Figure 6:**
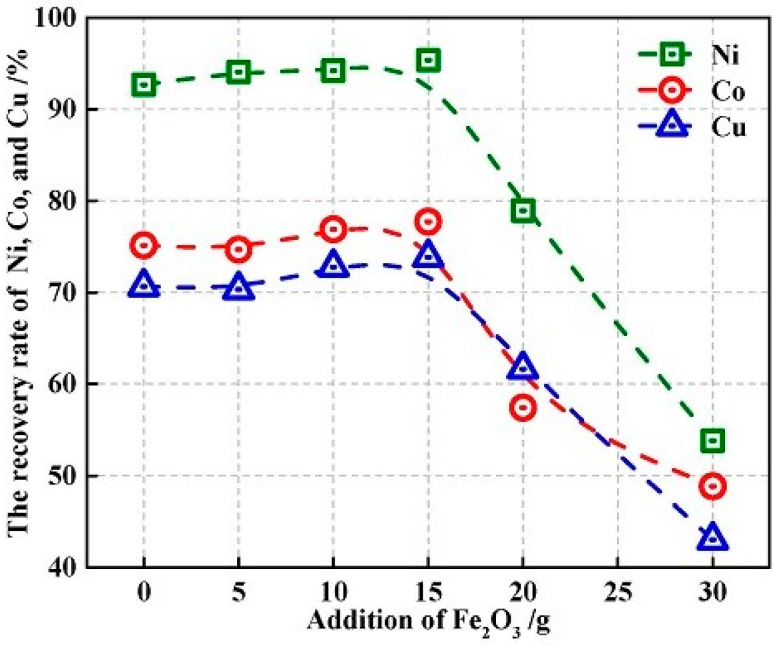
Addition of Fe_2_O_3_ versus metals recovery rate.

Figure 6 shows the Ni, Co and Cu recovery rates change with different addition of Fe_2_O_3_. With the Fe_2_O_3_ addition increased in the range of 0–15.0 g, the metals recovery rates increased. However, with the addition of Fe_2_O_3_ further increasing from 15.0 to 40.0 g, the metals recovery rate declined sharply. According to the phase diagram of FeO-Fe_2_O_3_-SiO_2_, the slag will melt very well at 1500 °C, while the phase diagram of Fe-Ni-Co-Cu-S shows that the decrease of sulfur content will make the liquidus temperature of metal increase significantly [45,46,47,48,49]. With the increase of Fe_2_O_3_ addition, the sulfur content in metal decreased, causing the high liquidus temperature of metal and poor separation of metal from slag. Therefore, although the removal of sulfur from slag promotes the reduction of oxides, the liquidus temperature of metal obtained increases simultaneously. The optimum addition of Fe_2_O_3_ was 15.0 g, while the desulfurization rate was 36.82% and the Ni, Co and Cu recovery rate were 95.33%, 77.73%, and 73.83%, respectively.

## 4. Conclusions

The Fe_2_O_3_ was used to remove sulfur firstly in the recovery of Ni, Co and Cu from the nickel converter slag as a weak oxidant due to the peroxidation of the molten slag with blowing air in the smelting oxidation-reduction process of nickel converter slag. The theoretical analysis and tubular furnace experiments were carried out to investigate the effect of desulfurization with Fe_2_O_3_ on the production process. The main conclusions are as following:

(1) the liquidus temperature decreased with the addition of Fe_2_O_3_ being in the range of 0–6 wt.%. however, the liquidus temperature began to increase for a Fe_2_O_3_ content of 6–40 wt.%. In addition, the desulfurization rate increased with the addition of Fe_2_O_3_, where the growth was the most significant for Fe_2_O_3_ contents of 5–20%.
(2) According to the FactSage calculations, a higher desulfurization rate of the slag promoted conversion of sulfides into oxides, which significantly increased the activities of Cu_2_O, NiO, and Fe_2_O_3_ in the slag. However, activity of CoO and FeO was not significantly affected.
(3) Although the removal of sulfur from slag promotes the carbothermic reduction of oxides, the liquidus temperature of metal obtained increases simultaneously. The optimum addition of Fe_2_O_3_ was 15.0 g, while the desulfurization rate was 36.82% and the metal recovery rate of Ni, Co, and Cu were 95.33%, 77.73%, and 73.83%, respectively.

## Figures and Tables

**Table 1:** Experimental schemes for the desulphurization of initial nickel converter slag with Fe_2_O_3_.

No.	Initial Slag (g)	Fe_2_O_3_ (g)	Final Slag (g)	Composition of Slag (wt.%)						
				Fe	FeO	Ni	Co	Cu	S	Al_2_O_3_
1	100.0	0.0	99.4	43.3	52.82	3.52	0.60	0.94	1.50	1.26
2	100.0	5.0	103.6	44.7	53.91	3.36	0.57	0.90	1.41	1.52
3	100.0	10.0	108.0	46.1	53.21	3.22	0.55	0.86	1.03	1.83
4	100.0	15.0	112.4	47.4	53.79	3.10	0.53	0.83	0.86	1.57
5	100.0	20.0	116.9	48.4	54.21	2.97	0.51	0.80	0.59	1.76
6	100.0	30.0	126.5	50.3	54.48	2.75	0.47	0.74	0.40	1.35
7 *	100.0	40.0	135.6	51.9	55.19	2.57	0.44	0.69	0.33	1.41

* With the poor separation of slag and alloy in Experiment No. 7, the alloy contains lots of slag.

**Table 2:** Experimental schemes for the reduction of the desulfurized slag with carbon powder.

No.	Initial Slag (g)	Carbon Powder (C/O = 0.2) (g)	Mass of Alloy (g)	Composition of Alloy (wt.%)			
				Ni	Co	Cu	S
1	49.7	1.04	6.42	25.25	3.49	5.14	2.92
2	51.8	1.11	6.39	25.62	3.45	5.13	2.55
3	54.0	1.21	6.94	23.60	3.29	4.87	1.58
4	56.2	1.30	7.19	23.10	3.22	4.79	1.20
5	58.5	1.38	6.23	22.01	2.75	4.63	0.77
6	63.2	1.56	4.65	20.11	3.12	4.33	0.45
7 *	67.8	1.73	−	−	−	−	−

* With the poor separation of slag and alloy in Experiment No. 7, the alloy contains lots of slag.
